# Supplementary material for: Isoform-resolved transcriptome of the human preimplantation embryo
Source: Nat Commun. 2023 Oct 30;14:6902. doi: 10.1038/s41467-023-42558-y (PMC10616205; doi:10.1038/s41467-023-42558-y)
Supplement: Supplementary file 1 — Supplementary Information [file 41467_2023_42558_MOESM1_ESM.pdf]

A - Illumina RNA-Seq quality control

Developmental stage	Samples	Average reads (millions)	Read range (millions)	Read length (paired-end)	GC%	Uniquely mapped
1C	13	57.1	24.4-93.5	100bp (n=3)<br>125bp (n=10)	45%-49%	82.2%-91.4%
2C	13	60.6	30.4-87.9	100bp (n=7)<br>125bp (n=6)	44.5%-48%	81.8%-91.8%
4C	16	59.2	28.9-112.7	100bp (n=5)<br>125bp (n=11)	45%-49%	61.7%-91.3%
8C	15	58.4	35.8-80.1	100bp (n=6)<br>125bp (n=9)	45%-48%	78.1%-89.5%
morula	3	36.4	9.9-57	125 (n=3)	46.5%-48%	80.3%-85.7%
blastocyst	13	67.3	36-81.6	100bp (n=8)<br>125bp (n=5)	44.5%-49%	78%-89.5%

B - PacBio IsoSeq quality control

Sample	Sequences	Average length (bp)	GC%	Uniquely mapped
human_2PN_1	287,888	1307.4	48%	99.97%
human_2PN_4	1,445,796	1388.8	45%	99.97%
human_2PN_EMB1	2,268,664	1578.3	46%	99.99%
human_2C_EMB2	348,373	1352.2	46%	99.94%
human_4C_EMB3	596,282	1269.5	46%	99.94%
human_4C_EMB4	1,771,779	1227.9	46%	99.94%
human_8C_EMB5	656,705	987.5	46%	99.95%
human_morula_MOR	2,193,063	1348.7	45%	99.95%
human_blastocyst_EMB6	331,247	490.0	50%	95.44%
human_merged_TD01673	223,237	1563.4	44%	99.69%

C

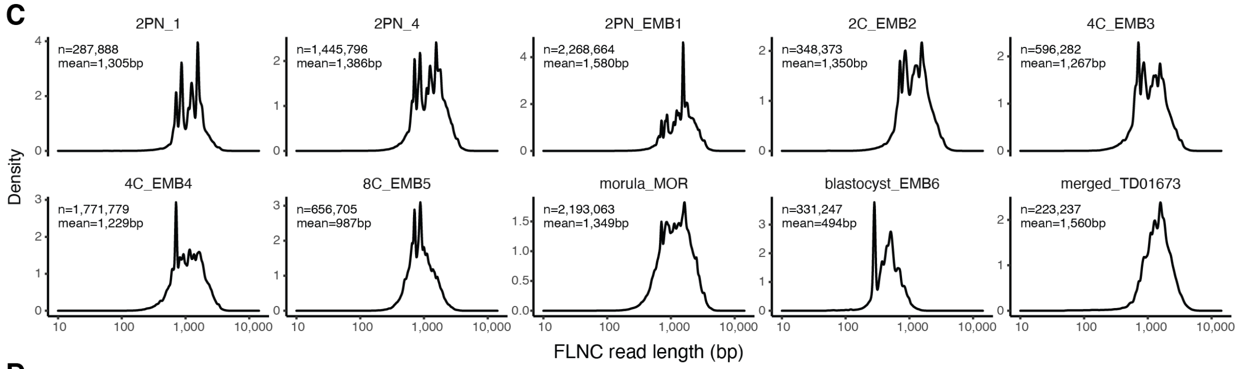

D

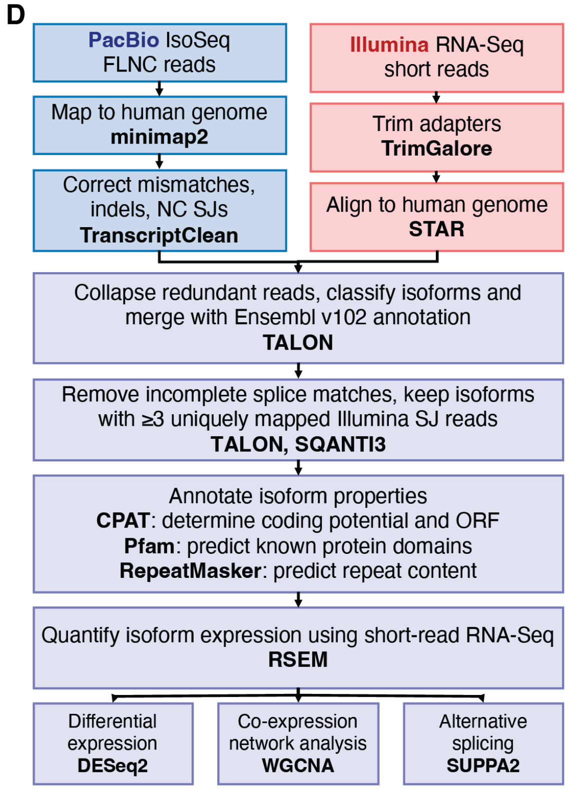

E

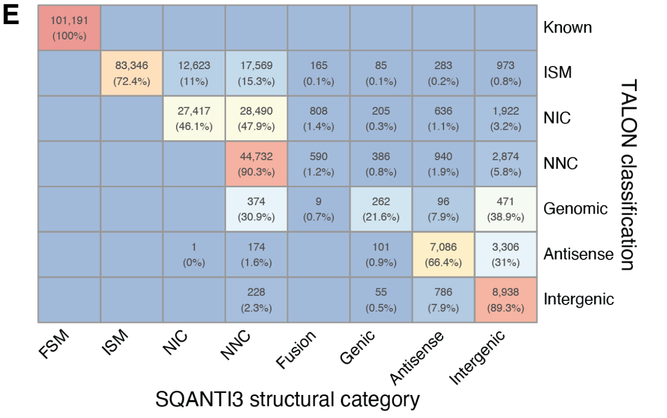

F

TALON class	SQANTI3 class	Applied class	Comments
ISM	Any other	ISM	All TALON ISMs are removed from transcriptome, irrespective of SQANTI3 class
NIC	NNC	NNC	Some isoforms are misannotated by TALON and correctly called by SQANTI3
Genomic	Any other	NNC	TALON "genomic" transcripts with SJ support are annotated as NNC, as they often share similar model structures
Antisense	Intergenic	Intergenic	Isoforms are labeled as "antisense" even if they are antisense to novel intergenic isoforms
Intergenic	Antisense	Antisense	Isoforms which are antisense to known genes, but only overlap 1bp

G **H** **I**

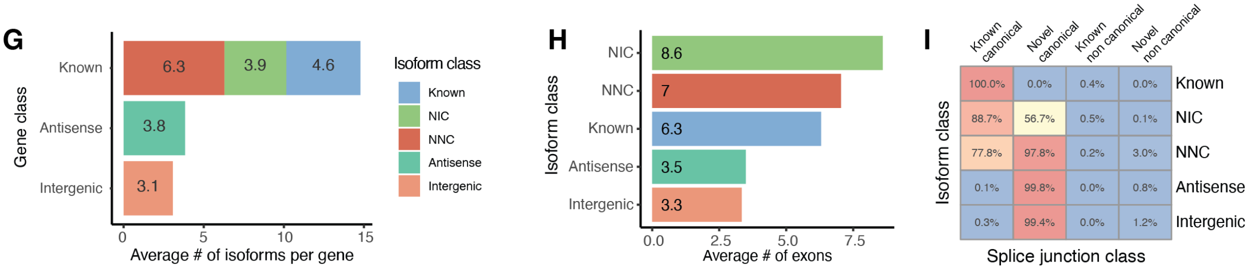

Supplementary Figure 1. Quality control and overview of the transcriptome generation pipeline.

A. Quality control of Illumina short-read sequencing data.
B. Quality control of PacBio long-read sequencing data.
C. Distribution of FLNC read lengths across PacBio replicates.
D. Workflow of the pipeline used to generate the novel reference transcriptome, and downstream analysis steps.
E. Comparison between transcript annotations generated by TALON (on rows) and SQANTI3 (on columns). Percentage values indicated are calculated for each row.
F. Table depicting the transcript class applied in cases where TALON and SQANTI3 generate conflicting classifications for a given isoform.
G. Average number of isoforms per gene, grouped by gene and isoform class.
H. Average number of exons for each isoform class.
I. Percentage of isoforms in each class displaying known or novel, canonical or noncanonical splice junctions.

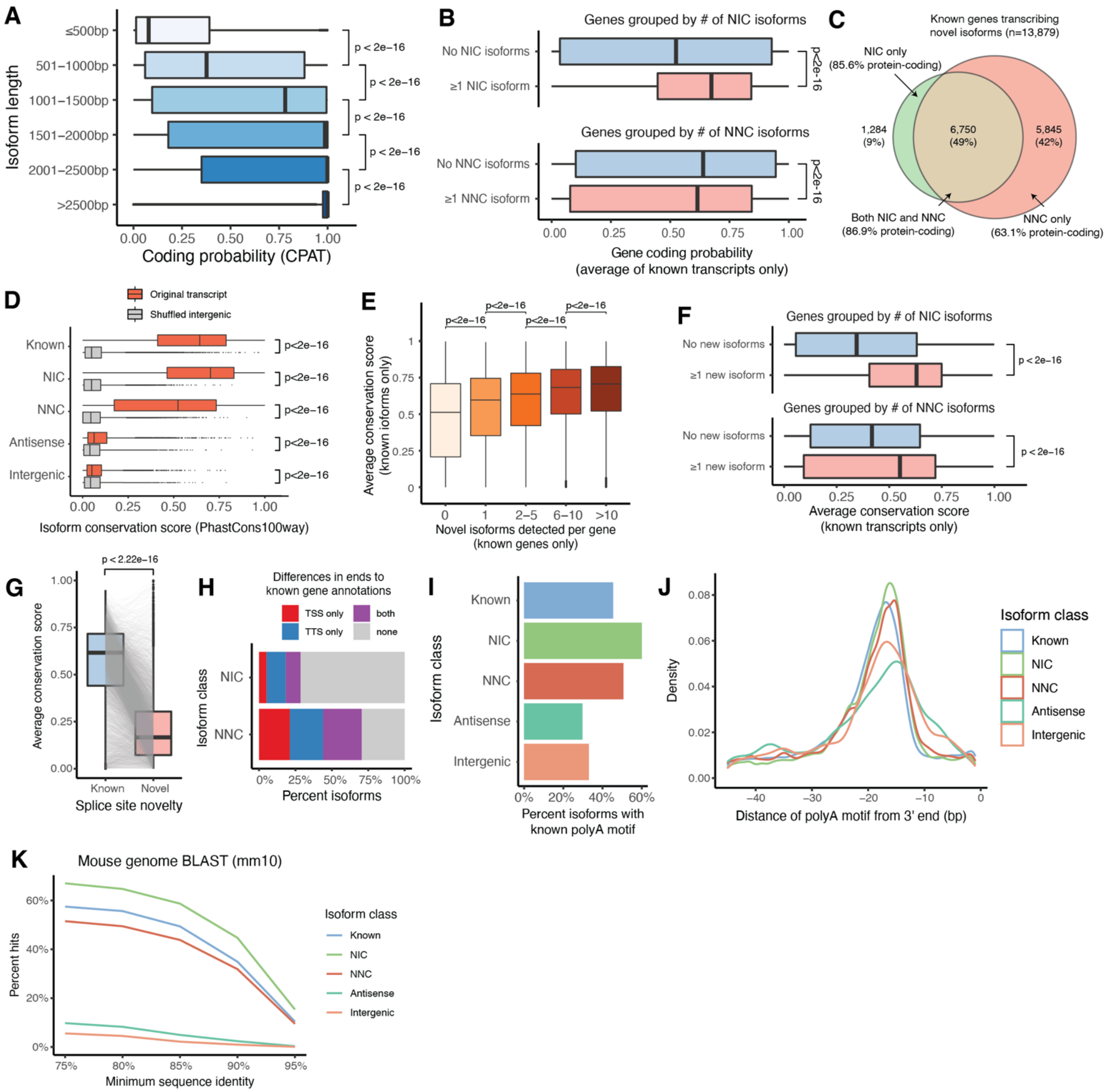

Supplementary Figure 2. Protein-coding potential and evolutionary conservation of the novel isoform-resolved transcriptome.

A. Distribution of predicted coding probabilities per isoform (from CPAT), grouped by isoform length. P-values were calculated using unpaired, two-sided Wilcoxon Rank Sum test, adjusted using the Benjamini-Hochberg method (≤500bp, n=14,115; 501-1000bp, n=64,539; 1001-1500bp, n=40,839; 1501-2000bp, n=32,978; 2001-2500bp, n=23,408; >2500bp, n=37,994).
B. Distribution of average gene coding probabilities, grouped according to the number of NIC and NNC isoforms transcribed. Only known transcripts are used for this calculation. P-values were calculated using unpaired, two-sided Wilcoxon Rank Sum test, adjusted using the Benjamini-Hochberg method (no NIC isoforms, n=12,758; ≥1 NIC isoform, n=8,034; no NNC isoforms, n=8,197; ≥1 NNC isoform, n=12,595).
C. Overlap between known genes transcribing at least one NIC isoform, one NNC isoform, or both novel isoform classes, and the percentage of protein-coding genes for each group.

D. Distribution of average isoform conservation scores (PhastCons100way) for each isoform class, compared to a background distribution generated by randomly shuffling transcript models in intergenic space. P-values were calculated using unpaired, two-sided Wilcoxon Rank Sum test, adjusted using the Benjamini-Hochberg method (sample sizes from **Figure 1C**).
E. Distribution of average conservation scores (PhastCons100way) of known genes, grouped by the number of novel isoforms detected per gene. P-values were calculated using unpaired, two-sided Wilcoxon Rank Sum test, adjusted using the Benjamini-Hochberg method (0 novel isoforms, n=6,913; 1 novel isoform, n=3,180; 2-5 novel isoforms, n=4,991; 6-10 novel isoforms, n=2,597; >10 novel isoforms, n=3,111).
F. Distribution of average conservation scores (PhastCons100way) of genes, grouped according to the number of NIC and NNC isoforms transcribed. Only known transcripts are used for this calculation. P-values were calculated using unpaired, two-sided Wilcoxon Rank Sum test, adjusted using the Benjamini-Hochberg method (sample sizes from **Suppl. Figure 2B**).
G. Boxplot displaying average conservation scores (PhastCons100way) of known and novel splice sites, for each gene. Average scores were calculated at genomic intervals ±10bp of each splice site. Only genes with at least 3 known and 3 novel splice sites were used (n=5,717). Known and novel splice sites from the same gene are connected by grey lines. P-value was calculated using paired, two-sided Wilcoxon Rank Sum test.
H. Percentage of NIC and NNC isoforms with different TSS, TSS or both, when compared to the known ends of the source gene.
I. Percentage of isoforms with a known polyA motif detected within 50bp of its 3’ end, grouped by isoform class.
J. Distribution of distances of detected polyA motifs from the isoform 3’ end across isoform classes, for isoforms with a detected motif.
K. Percentage of isoforms in each class classified as hits to the mouse genome (mm10) by BLAST, across increasing values of minimum sequence identity. To be classified as a hit, isoforms are further required to match the genome sequence for >100bp.

For the box plots in Suppl. Figure 2A-B, D-G, box limits extend from the 25th to 75th percentile, while the middle line represents the median. Whiskers extend to the largest value no further than 1.5 times the inter-quartile range (IQR) from each box hinge. Points beyond the whiskers are outliers.

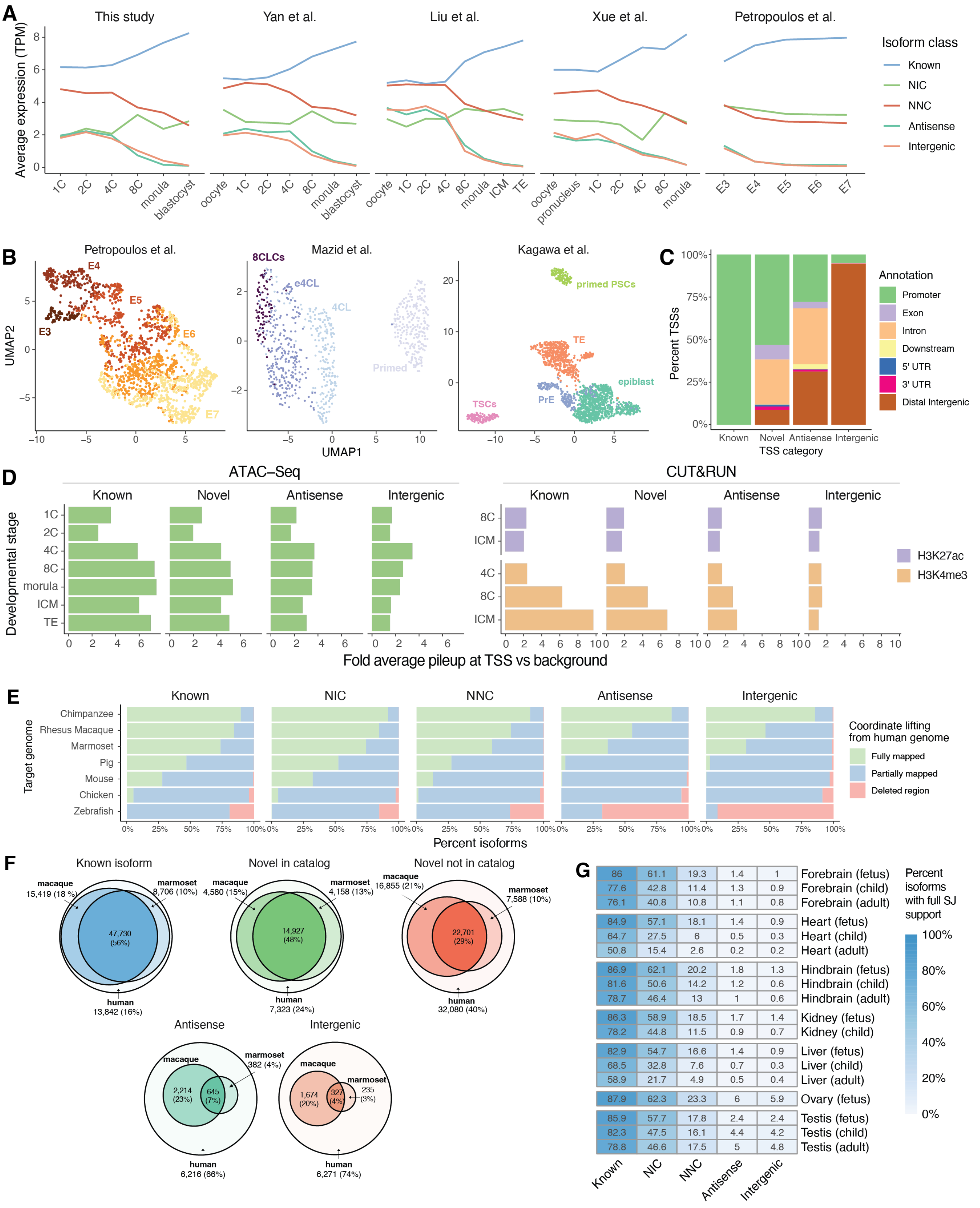
A
This study
Yan et al.
Liu et al.
Xue et al.
Petropoulos et al.
Average expression (TPM)
Isoform class
Known
NIC
NNC
Antisense
Intergenic
1C 2C 4C 8C morula blastocyst
oocyte 1C 2C 4C 8C morula blastocyst
oocyte 1C 2C 4C 8C morula ICM TE
oocyte pronucleus 1C 2C 4C 8C morula
E3 E4 E5 E6 E7
B
Petropoulos et al.
Mazid et al.
Kagawa et al.
UMAP1
UMAP2
E3 E4 E5 E6 E7
8CLCs
e4CL
4CL
Primed
primed PSCs
TE
epiblast
PrE
TSCs
C
Percent TSSs
TSS category
Known Novel Antisense Intergenic
Annotation
Promoter
Exon
Intron
Downstream
5' UTR
3' UTR
Distal Intergenic
D
ATAC–Seq
CUT&RUN
Known
Novel
Antisense
Intergenic
Developmental stage
1C 2C 4C 8C morula ICM TE
8C ICM
4C 8C ICM
H3K27ac
H3K4me3
Fold average pileup at TSS vs background
E
Known
NIC
NNC
Antisense
Intergenic
Target genome
Chimpanzee
Rhesus Macaque
Marmoset
Pig
Mouse
Chicken
Zebrafish
Percent isoforms
Coordinate lifting from human genome
Fully mapped
Partially mapped
Deleted region
F
Known isoform
macaque 15,419 (18 %)
marmoset 8,706 (10%)
47,730 (56%)
human 13,842 (16%)
Novel in catalog
macaque 4,580 (15%)
marmoset 4,158 (13%)
14,927 (48%)
human 7,323 (24%)
Novel not in catalog
macaque 16,855 (21%)
marmoset 7,588 (10%)
22,701 (29%)
human 32,080 (40%)
Antisense
macaque
marmoset 382 (4%)
2,214 (23%)
645 (7%)
human 6,216 (66%)
Intergenic
macaque
marmoset 235 (3%)
1,674 (20%)
327 (4%)
human 6,271 (74%)
G
Known NIC NNC Antisense Intergenic
86 61.1 19.3 1.4 1 Forebrain (fetus)
77.6 42.8 11.4 1.3 0.9 Forebrain (child)
76.1 40.8 10.8 1.1 0.8 Forebrain (adult)
84.9 57.1 18.1 1.4 0.9 Heart (fetus)
64.7 27.5 6 0.5 0.3 Heart (child)
50.8 15.4 2.6 0.2 0.2 Heart (adult)
86.9 62.1 20.2 1.8 1.3 Hindbrain (fetus)
81.6 50.6 14.2 1.2 0.6 Hindbrain (child)
78.7 46.4 13 1 0.6 Hindbrain (adult)
86.3 58.9 18.5 1.7 1.4 Kidney (fetus)
78.2 44.8 11.5 0.9 0.7 Kidney (child)
82.9 54.7 16.6 1.4 0.9 Liver (fetus)
68.5 32.8 7.6 0.7 0.3 Liver (child)
58.9 21.7 4.9 0.5 0.4 Liver (adult)
87.9 62.3 23.3 6 5.9 Ovary (fetus)
85.9 57.7 17.8 2.4 2.4 Testis (fetus)
82.3 47.5 16.1 4.4 4.2 Testis (child)
78.8 46.6 17.5 5 4.8 Testis (adult)
Percent isoforms with full SJ support
100% 80% 60% 40% 20% 0%

Supplementary Figure 3. QC and analysis of integrated publicly available transcriptomic and epigenomic datasets.

A. Line plots displaying average normalized expression levels (TPM – transcripts per million) for each isoform class across human preimplantation developmental stages.
B. UMAP plots generated by quantifying gene expression of the orthogonal, previously published datasets to the novel isoforms in the isoform-resolved transcriptome presented in this study.
C. Genomic annotation of novel TSS categories compared to known transcripts in the Ensembl v102 annotation.
D. Average fold enrichment of ATAC-Seq and CUT&RUN signal within ±500bp of each TSS category across developmental stages, compared to background. All conditions are significantly more highly enriched than background ($p<2e-16$, Wilcoxon rank-sum test, unpaired and two-sided test).
E. Percentage of isoforms whose genomic regions fully map, partially map or do not map to a selection of target vertebrate genomes using liftOver, grouped by isoform class.
F. Overlap of isoforms fully mapping to the macaque and marmoset genomes, and detected in embryonic short-read RNA-Seq datasets for each species.
G. Percentage of isoforms for each structural class with full short read RNA-Seq splice junction support across tissues and developmental timepoints (data from Mazin et al.).

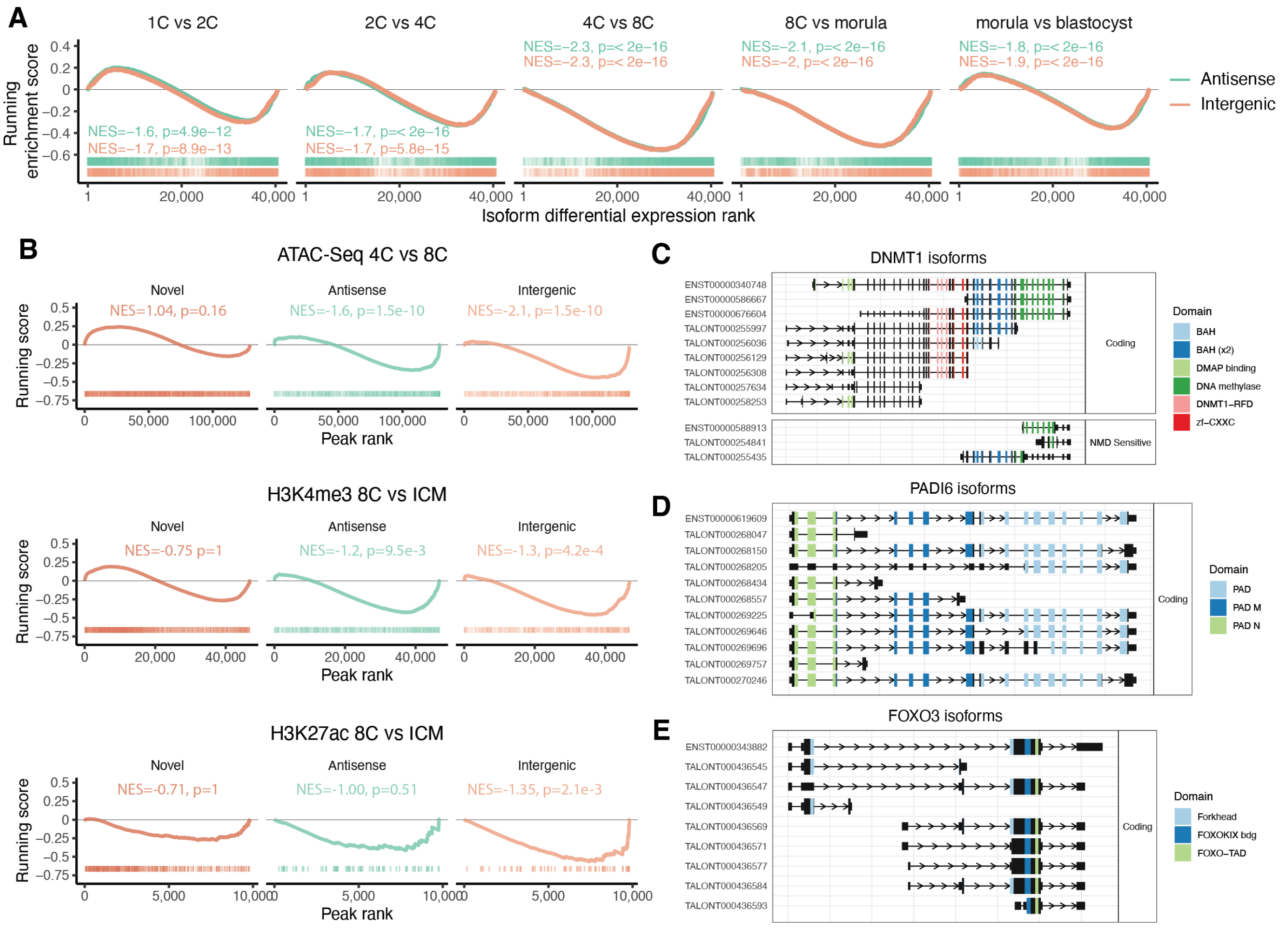

Supplementary Figure 4. Dynamics of novel isoform expression throughout development.

A. Enrichment analysis of novel antisense and intergenic isoforms along isoform-level differential expression signatures across developmental stages. Isoforms are ranked by most strongly upregulated (lowest rank, on left of each subplot) to most strongly downregulated (highest rank, on right). Vertical bars represent the position of genes of each class within the ranking. Also reported are p-values and normalized enrichment scores (NES).
B. Line plots displaying enrichment of ATAC-Seq, H3K4me3 and H3K27ac peaks overlapping novel TSSs categories in selected datasets and developmental stage transitions
C. to E. Selected isoforms for DNMT1, PADI6 and FOXO3, including both known isoforms from the Ensembl v102 transcriptome release (named ENST*), and novel isoforms detected from the long-read sequencing data (named TALONT*). Further displayed are the predicted protein-coding status, NMD sensitivity, ORF sequences, and known domains.

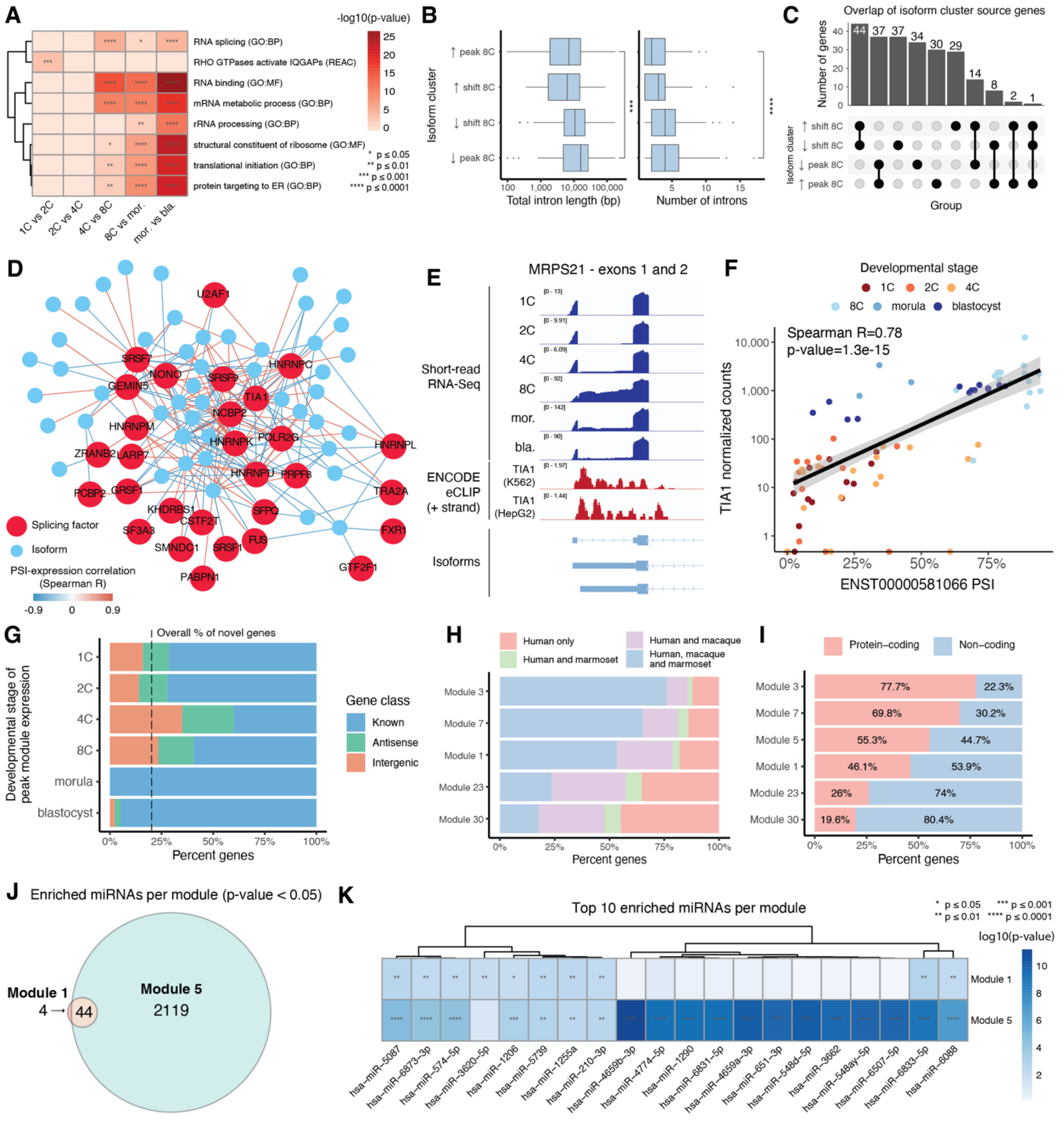

Supplementary Figure 5. Biological properties of alternatively spliced isoforms and co-expressed gene modules in the novel isoform-resolved transcriptome.

A. Heatmap displaying enrichment results of genes undergoing AS at each developmental stage transition. P-values were calculated using g:Profiler (see Methods for details).

B. Distribution of total intron lengths and number of introns across isoform clusters defined in **Figure 5E**. P-values were calculated using unpaired, two-sided Wilcoxon Rank Sum test, adjusted using the Benjamini-Hochberg method (for total intron length comparison, p=3.98e-4; for number of intron comparison, p=4.21e-5).

C. UpSet plot displaying the number of genes transcribing isoforms assigned to one or multiple combinations of isoform clusters.
D. Network displaying pairs of splicing factors (SFs, red) and isoforms (blue). Edges indicate pairs where the correlation between SF expression and isoform inclusion is ≥0.75 (Spearman R), and the SF has at least one eCLIP binding peak overlapping the isoform sequence in the same strand (data from ENCODE).
E. Genome browser snapshot of the first two exons of MRPS21, alongside short-read RNA-Seq expression for each developmental stage, and ENCODE eCLIP pileups of TIA1 in K562 and HepG2 cells.
F. Correlation between relative inclusion of ENST00000581066 (intron retention isoform) and TIA1 across short-read RNA-Seq embryo samples. Colors indicate developmental stage of each embryo.
G. Bar chart displaying the gene composition of modules, grouped according to which developmental stage the modules reach peak expression. The vertical line displays the overall percentage of novel genes in the novel transcriptome.
H. Percentage of genes detected in human, macaque and marmoset preimplantation embryo RNA-Seq datasets for selected modules.
I. Bar plot displaying the percentage of protein-coding and non-coding genes for selected modules, as defined by having at least one predicted protein-coding isoform.
J. Venn diagram displaying the number of significantly enriched miRNAs for each module as predicted by overrepresentation analysis ($p<0.05$, Benjamini-Hochberg correction). miRNA-gene binding was predicted using miRanda.
K. Heatmap displaying log10(p-value) and statistical significance between miRNAs and gene modules as predicted by overrepresentation analysis (using clusterProfiler, see Methods). The top 10 enriched miRNAs for each module are displayed.

For the box plots in Suppl. Figure 5B, box limits extend from the 25th to 75th percentile, while the middle line represents the median. Whiskers extend to the largest value no further than 1.5 times the inter-quartile range (IQR) from each box hinge. Points beyond the whiskers are outliers.

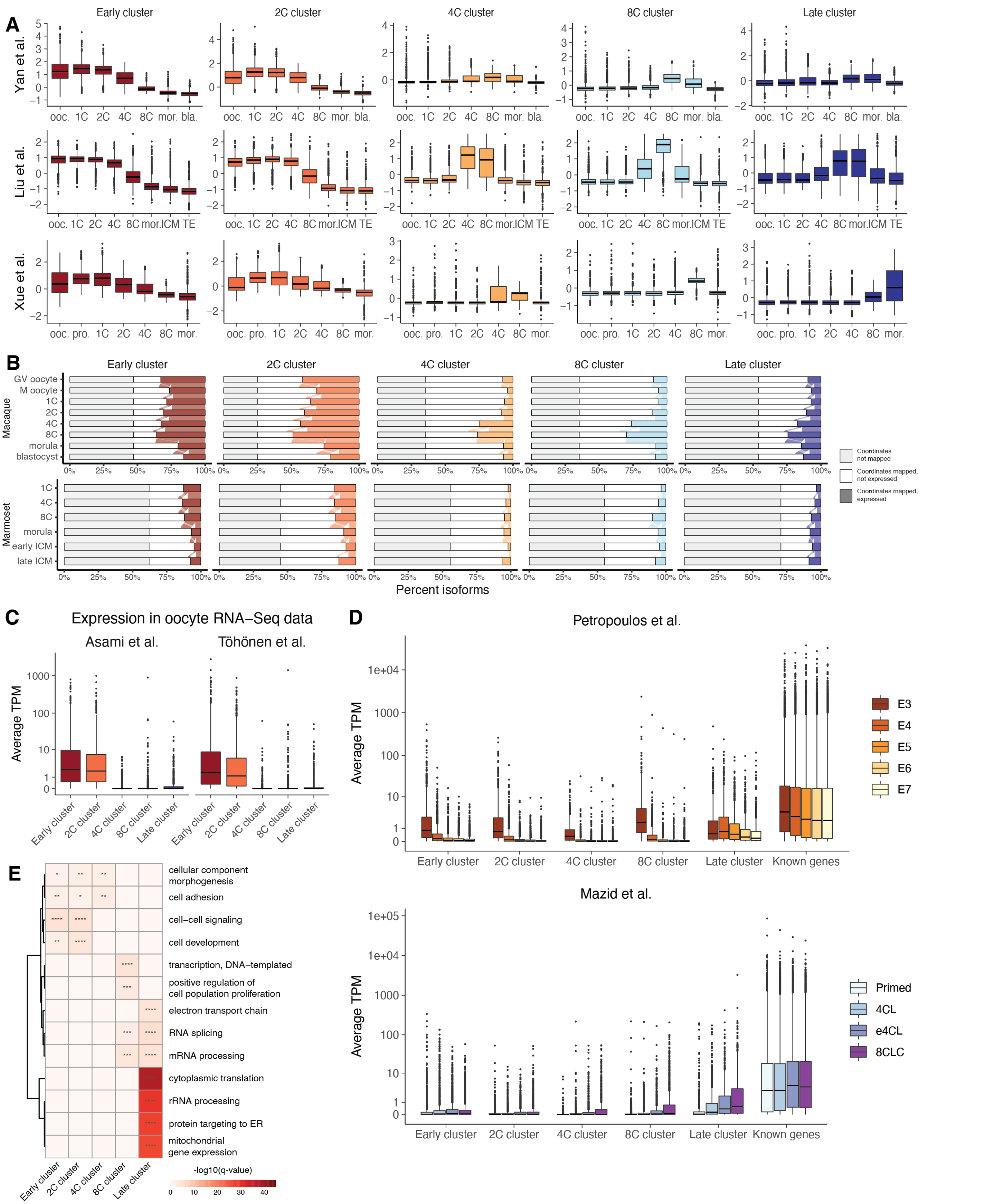
A
Early cluster
2C cluster
4C cluster
8C cluster
Late cluster
Yan et al.
Liu et al.
Xue et al.
B
Macaque
Marmoset
Coordinates not mapped
Coordinates mapped, not expressed
Coordinates mapped, expressed
Percent isoforms
C
Expression in oocyte RNA–Seq data
Asami et al.
Töhönen et al.
Average TPM
D
Petropoulos et al.
Mazid et al.
Known genes
E3
E4
E5
E6
E7
Primed
4CL
e4CL
8CLC
E
cellular component morphogenesis
cell adhesion
cell–cell signaling
cell development
transcription, DNA–templated
positive regulation of cell population proliferation
electron transport chain
RNA splicing
mRNA processing
cytoplasmic translation
rRNA processing
protein targeting to ER
mitochondrial gene expression
-log10(q-value)

Supplementary Figure 6. Expression patterns and associated biological pathways of novel gene clusters across publicly available transcriptomic datasets.

A. Box plots displaying normalized gene expression values (VST) of novel gene clusters in short-read RNA-Seq data from three integrated publicly available embryo short-read RNA-Seq studies (sample sizes for each stage shown in **Figure 1A**).
B. Percentage of genes in each cluster that map to the macaque and marmoset genomes, and are expressed across developmental stages in corresponding preimplantation short-read RNA-Seq datasets.
C. Box plots displaying average normalized expression (TPM – transcripts per million) of novel genes in oocyte RNA-Seq samples (data from Asami et al., Töhönen et al.) across novel gene clusters (sample sizes in from **Figure 7A**)
D. As above, but displaying expression data from Petropoulos et al. and Mazid et al. SmartSeq2 single-cell RNA-Seq data, grouped by cluster (sample sizes for each stage shown in **Figure 1A**).
E. Significantly enriched Gene Ontology: Biological Process terms for each novel gene cluster, calculated using g:Profiler. Results are calculated by performing an enrichment analysis of the top 1,000 most co-expressed genes for each cluster as determined using WGCNA.

For the box plots in Suppl. Figure 6A,C,D, box limits extend from the 25th to 75th percentile, while the middle line represents the median. Whiskers extend to the largest value no further than 1.5 times the inter-quartile range (IQR) from each box hinge. Points beyond the whiskers are outliers.

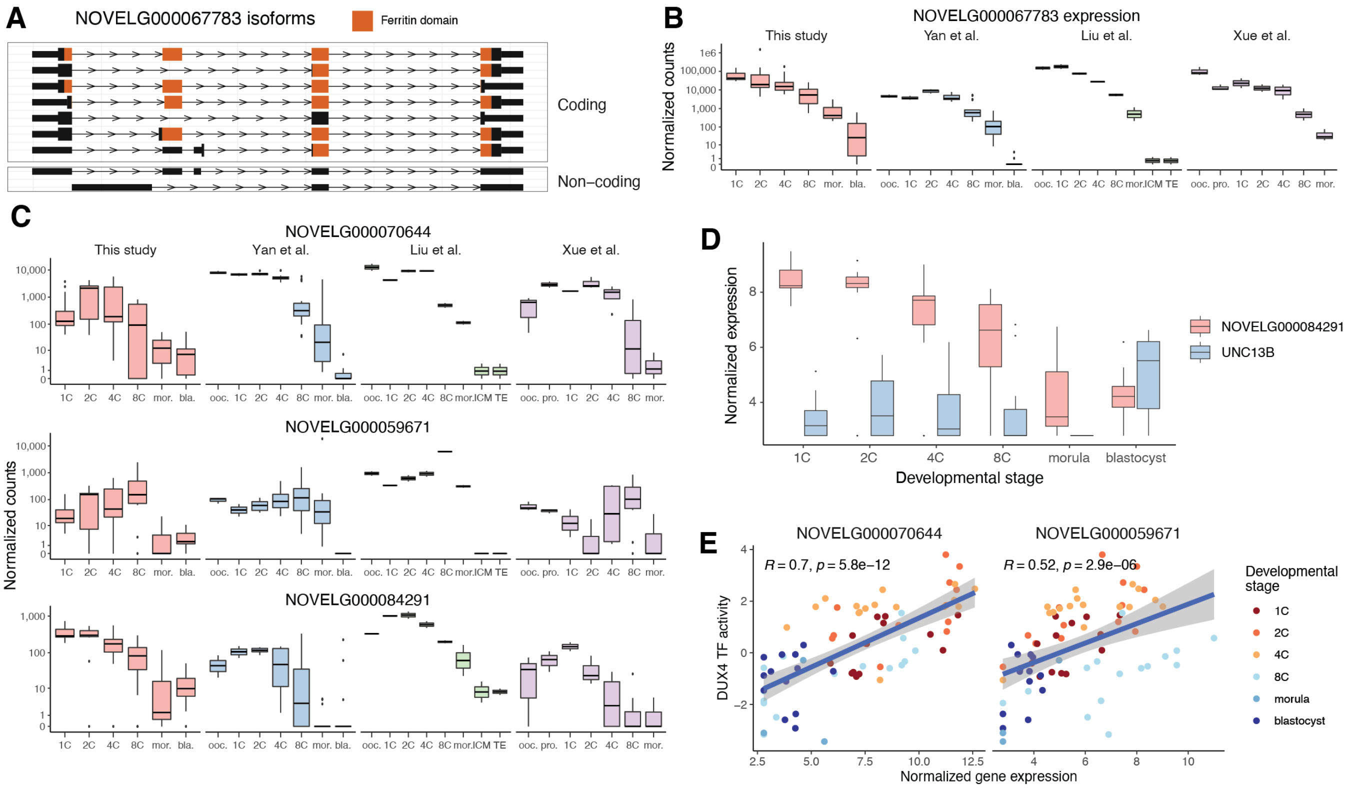

Supplementary Figure 7. Selected isoforms and expression of candidate novel genes from the isoform-resolved transcriptome.

A. Isoforms of NOVELG000067783, with predicted protein-coding status, ORF sequence, and protein domains highlighted
B. Normalized counts (DESeq2 size factors) of NOVELG000067783 in this study and publicly available human embryo short-read RNA-Seq studies (full sample sizes for each dataset available in Supplementary Data 6).
C. As above, but for NOVELG000084291, NOVELG000070644 and NOVELG000084291.
D. Normalized counts (VST) of NOVELG000084291 and its antisense neighbor UNC13B throughout developmental stages from short-read RNA-Seq data collected in this study.
E. Correlation between VIPER-inferred DUX4 activity and the expression of NOVELG000070644 and NOVELG000059671 across short-read RNA-Seq samples collected in this study. Points are colored by developmental stage. Also displayed are correlation statistics determined using Spearman's index.

For the box plots in Suppl. Figure 7B-D, box limits extend from the 25th to 75th percentile, while the middle line represents the median. Whiskers extend to the largest value no further than 1.5 times the inter-quartile range (IQR) from each box hinge. Points beyond the whiskers are outliers.

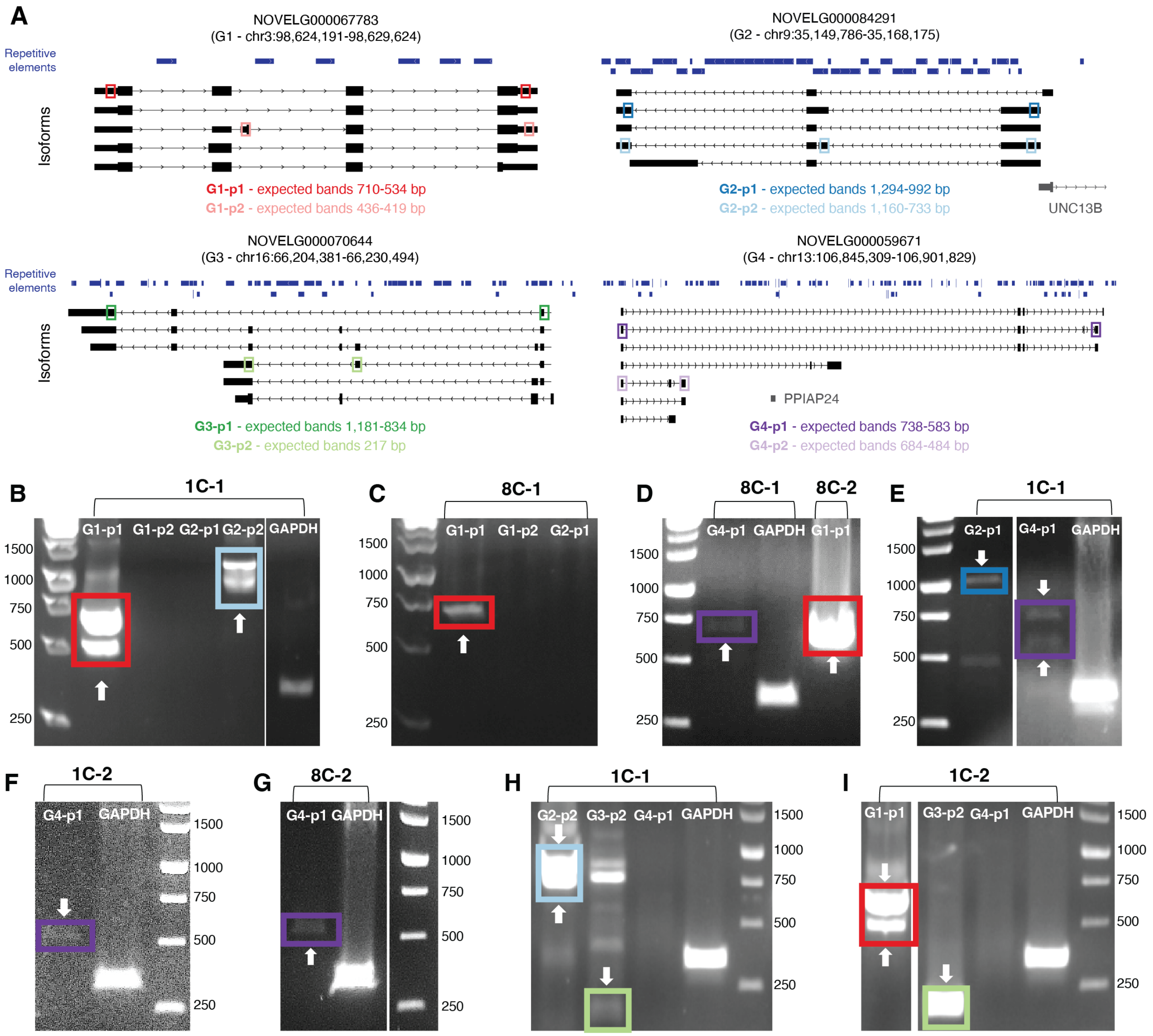

Supplementary Figure 8. PCR validation of candidate novel genes from the isoform-resolved transcriptome.

A. Isoform models and repetitive element annotations (RepeatMasker) for NOVELG000067783 (G1), NOVELG000084291 (G2), NOVELG000070644 (G3), and NOVELG000059671 (G4). Also highlighted are the genomic locations of the forward and reverse primer pairs with corresponding expected length of the PCR product. Note: the reverse primer for G2-p2 maps to both the last exon (light blue, left-most in the panel) and an internal exon, due to the presence of repetitive elements (LTR7); in-silico PCR does not predict other off-target effects. Full primer sequences are available in **Suppl Table 9**.

B. to I. Gel electrophoresis displaying PCR results from human preimplantation embryo cDNA samples, using primer pairs illustrated in **Suppl. Figure 8A**. Four separate samples were assessed: 1C-1 and 1C-2 (both independently generated by pooling three sets of separate 1C embryos), 8C-1 and 8C-2 (pooling four E3 embryos each).

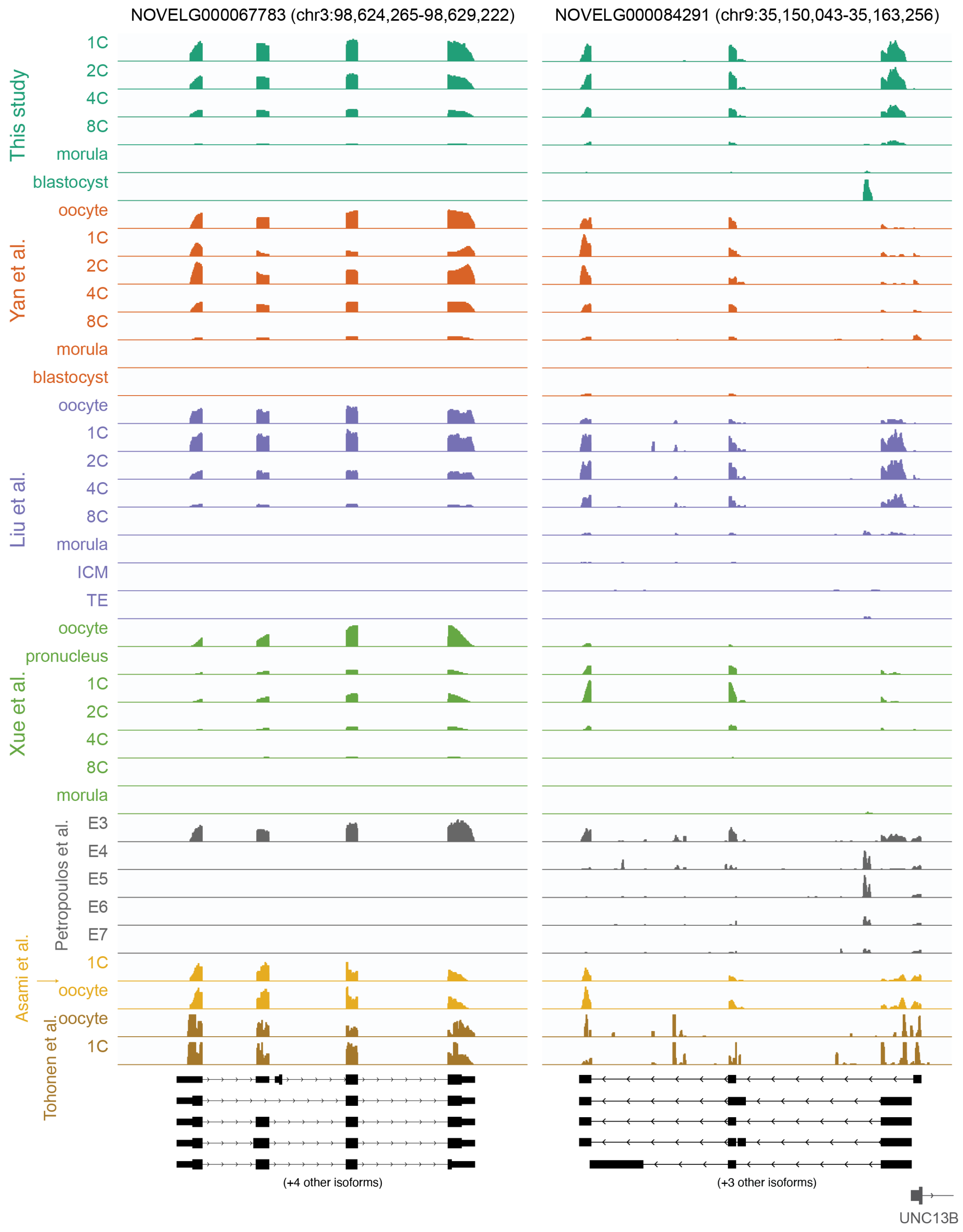
NOVELG000067783 (chr3:98,624,265-98,629,222)
NOVELG000084291 (chr9:35,150,043-35,163,256)
This study
1C
2C
4C
8C
morula
blastocyst
Yan et al.
oocyte
1C
2C
4C
8C
morula
blastocyst
Liu et al.
oocyte
1C
2C
4C
8C
morula
ICM
TE
Xue et al.
oocyte
pronucleus
1C
2C
4C
8C
morula
Petropoulos et al.
E3
E4
E5
E6
E7
Asami et al.
1C
oocyte
Tohonen et al.
oocyte
1C
(+4 other isoforms)
(+3 other isoforms)
UNC13B

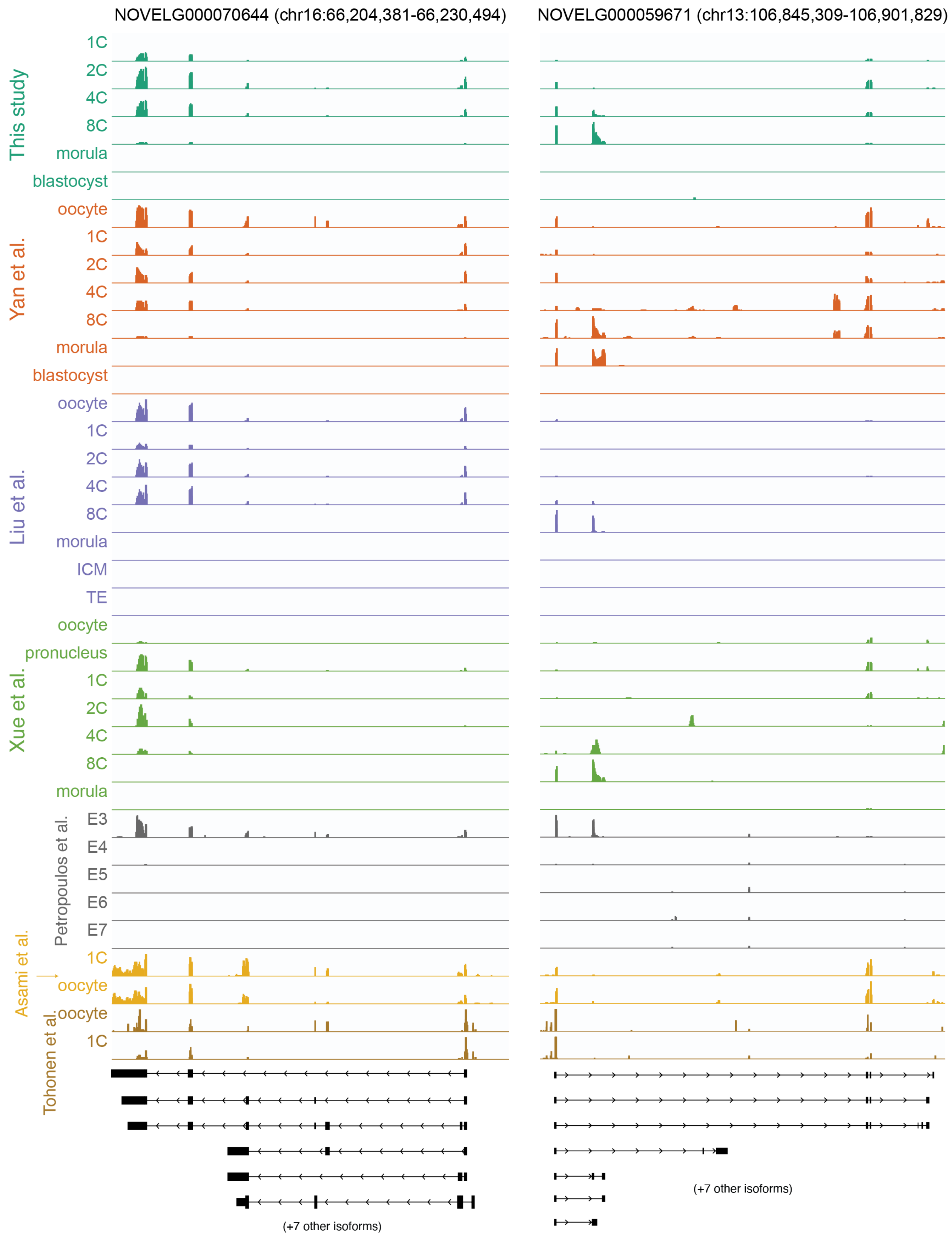
NOVELG000070644 (chr16:66,204,381-66,230,494)
NOVELG000059671 (chr13:106,845,309-106,901,829)
This study
1C
2C
4C
8C
morula
blastocyst
Yan et al.
oocyte
1C
2C
4C
8C
morula
blastocyst
Liu et al.
oocyte
1C
2C
4C
8C
morula
ICM
TE
Xue et al.
oocyte
pronucleus
1C
2C
4C
8C
morula
Petropoulos et al.
E3
E4
E5
E6
E7
Asami et al.
1C
oocyte
Tohonen et al.
oocyte
1C
(+7 other isoforms)
(+7 other isoforms)

Supplementary Figure 9. Short-read RNA-Seq data from independently published human preimplantation embryo studies shows support of all four novel genes highlighted. Genome browser tracks displaying novel genes, alongside average normalized pileups (RPKM) derived from short-read RNA-Seq data across developmental stages from independently published studies profiling human preimplantation embryos and oocytes.

Uncropped scans of blots in Supplementary Figure 8

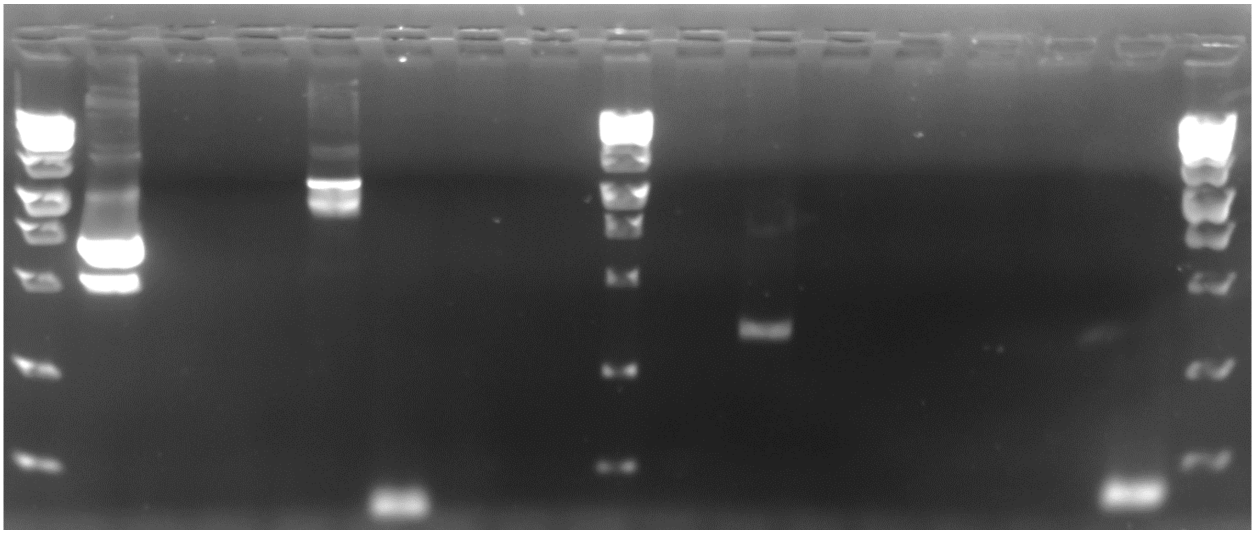

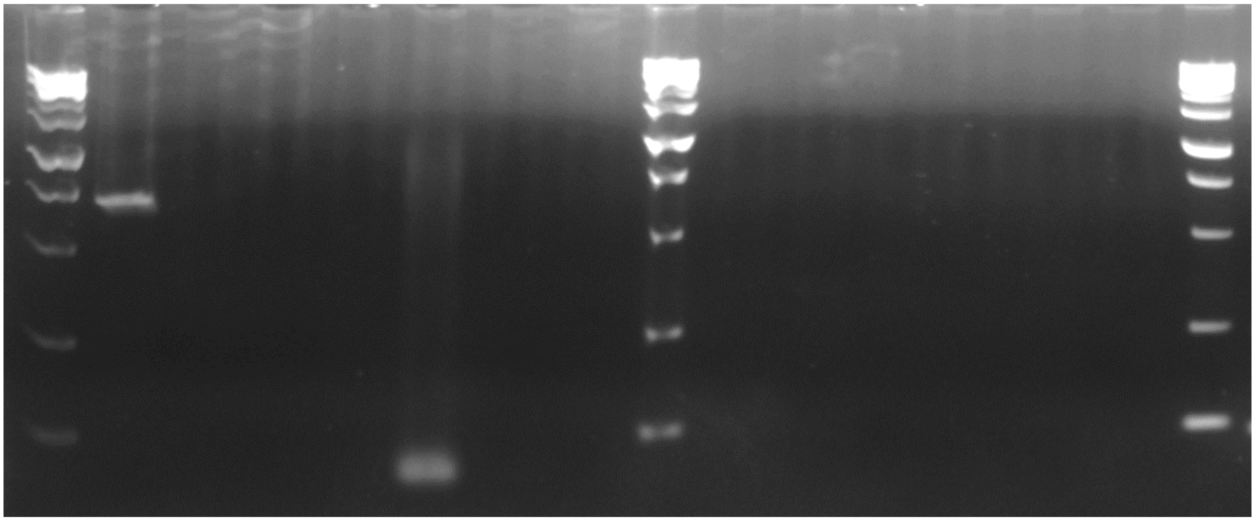

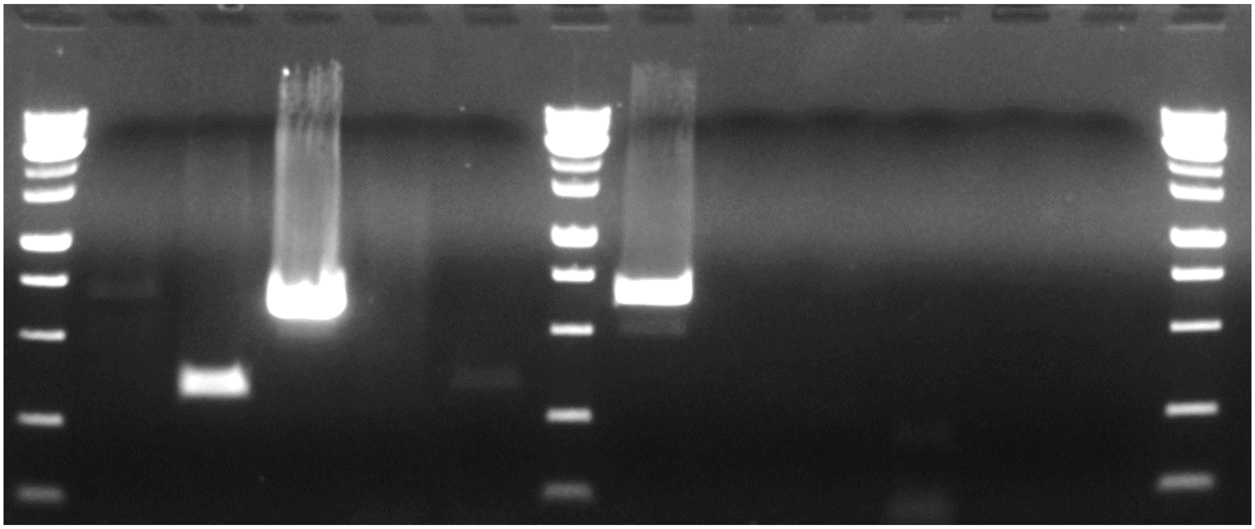

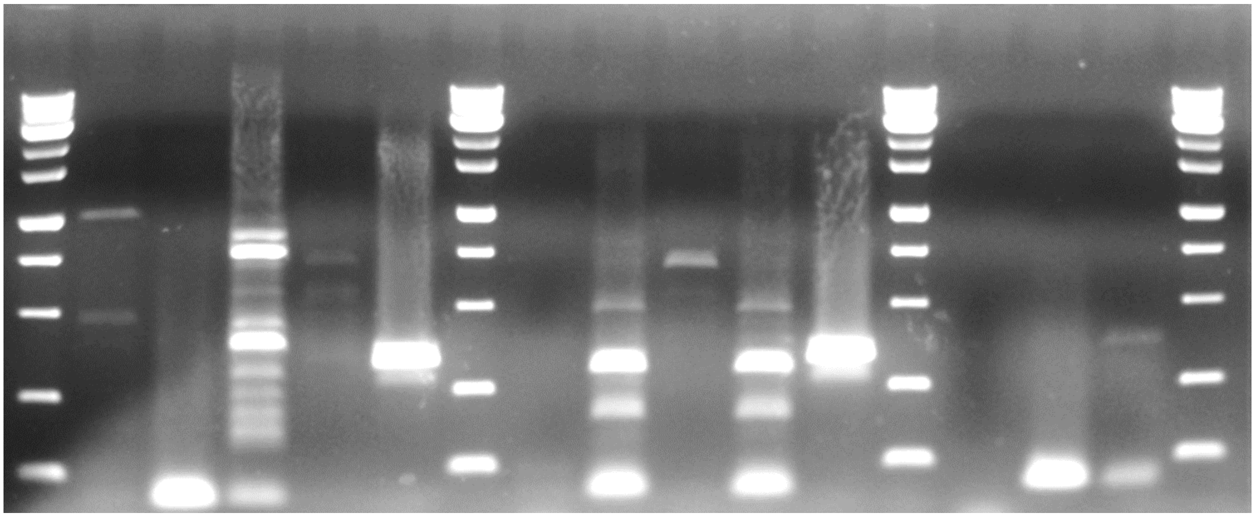
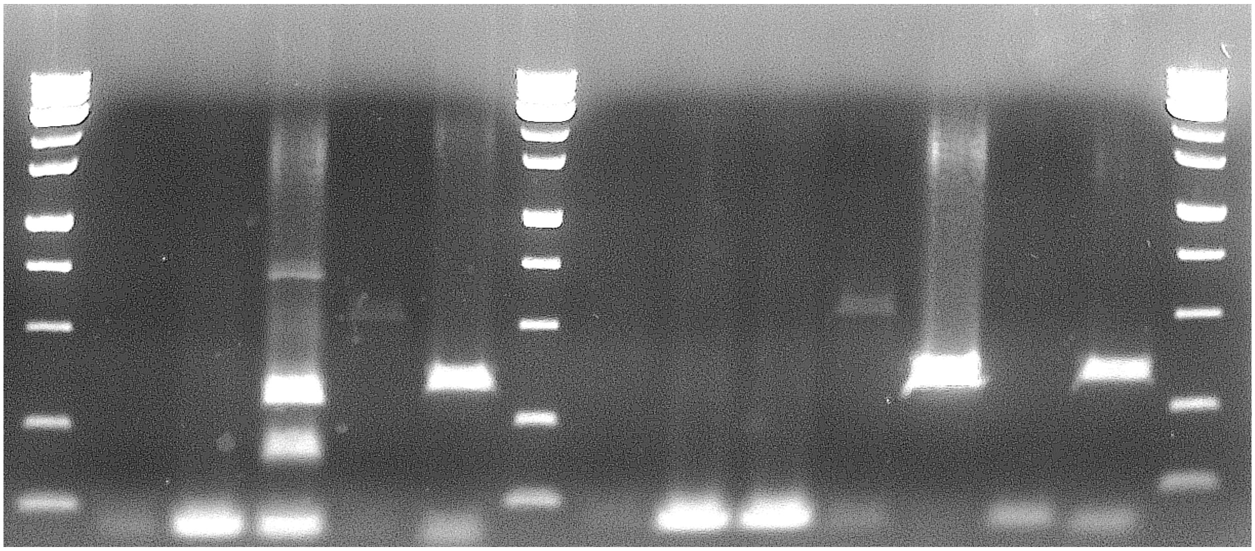
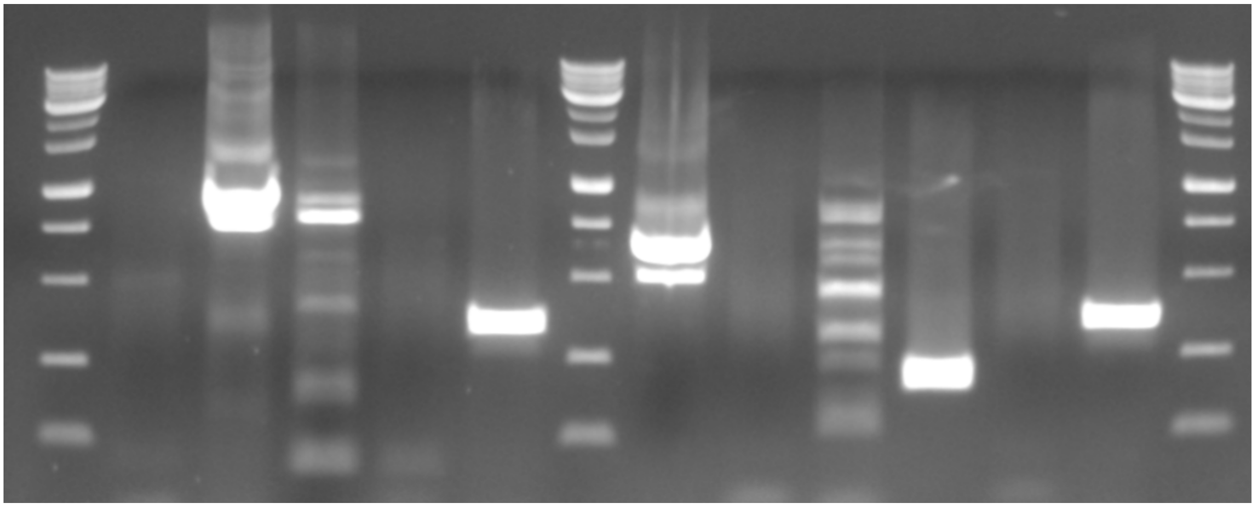

Supplementary Data Legends

- **Supplementary Data 1.** Overview of the human preimplantation embryos used in this study.
- **Supplementary Data 2.** Summary of isoform features, including structural categories, predicted coding probabilities, protein domain content, repetitive element integrations, and evolutionary conservation.
- **Supplementary Data 3.** Predicted isoform protein coding probabilities and open reading frame (ORF) locations, calculated using CPAT.
- **Supplementary Data 4.** Location of protein domains within ORFs of predicted coding isoforms, calculated using PfamScan.
- **Supplementary Data 5.** Predicted repetitive element integrations within isoforms, calculated using RepeatMasker.
- **Supplementary Data 6.** Summary and QC of integrated multi-omics, independently published embryo datasets.
- **Supplementary Data 7.** Known developmental genes displayed in **Figure 4D**, with relevant citations.
- **Supplementary Data 8.** Information on isoform cluster assignments (**Figure 5E**), isoform cluster-RBP correlations (**Figure 5I**), and the isoform-RBP network (**Suppl. Figure 5D**).
- **Supplementary Data 9.** Primers for PCR validation of novel genes (**Suppl. Figure 8**).
