# Supplementary material for: Isoform-resolved transcriptome of the human preimplantation embryo
Source: Nat Commun. 2023 Oct 30;14:6902. doi: 10.1038/s41467-023-42558-y (PMC10616205; doi:10.1038/s41467-023-42558-y)
Supplement: Supplementary file 3 — Description of Additional Supplementary Files [file 41467_2023_42558_MOESM3_ESM.pdf]

Description of additional supplementary files

Supplementary Data 1. Overview of the human preimplantation embryos used in this study.

Supplementary Data 2. Summary of isoform features, including structural categories, predicted coding probabilities, protein domain content, repetitive element integrations, and evolutionary conservation.

Supplementary Data 3. Predicted isoform protein coding probabilities and open reading frame (ORF) locations, calculated using CPAT.

Supplementary Data 4. Location of protein domains within ORFs of predicted coding isoforms, calculated using PfamScan.

Supplementary Data 5. Predicted repetitive element integrations within isoforms, calculated using RepeatMasker.

Supplementary Data 6. Summary and QC of integrated multi-omics, independently published embryo datasets.

Supplementary Data 7. Known developmental genes displayed in Figure 4D, with relevant citations.

Supplementary Data 8. Information on isoform cluster assignments (Figure 5E), isoform cluster-RBP correlations (Figure 5I), and the isoform-RBP network (Suppl. Figure 5D).

Supplementary Data 9. Primers for PCR validation of novel genes (Suppl. Figure 8).
