# Supplementary material for: Isoform-resolved transcriptome of the human preimplantation embryo
Source: Nat Commun. 2023 Oct 30;14:6902. doi: 10.1038/s41467-023-42558-y (PMC10616205; doi:10.1038/s41467-023-42558-y)
Supplement: Supplementary file 13 — Reporting Summary [file 41467_2023_42558_MOESM13_ESM.pdf]

nature portfolio

Corresponding author(s): Robert P Sebra, Dalit Ben-Yosef

Last updated by author(s): 10/04/2023

Reporting Summary

Nature Portfolio wishes to improve the reproducibility of the work that we publish. This form provides structure for consistency and transparency in reporting. For further information on Nature Portfolio policies, see our Editorial Policies and the Editorial Policy Checklist.

Statistics

For all statistical analyses, confirm that the following items are present in the figure legend, table legend, main text, or Methods section.

n/a	Confirmed	
☐	☒	The exact sample size (*n*) for each experimental group/condition, given as a discrete number and unit of measurement
☐	☒	A statement on whether measurements were taken from distinct samples or whether the same sample was measured repeatedly
☐	☒	The statistical test(s) used AND whether they are one- or two-sided<br>*Only common tests should be described solely by name; describe more complex techniques in the Methods section.*
☐	☒	A description of all covariates tested
☐	☒	A description of any assumptions or corrections, such as tests of normality and adjustment for multiple comparisons
☐	☒	A full description of the statistical parameters including central tendency (e.g. means) or other basic estimates (e.g. regression coefficient) AND variation (e.g. standard deviation) or associated estimates of uncertainty (e.g. confidence intervals)
☐	☒	For null hypothesis testing, the test statistic (e.g. *F*, *t*, *r*) with confidence intervals, effect sizes, degrees of freedom and *P* value noted<br>*Give P values as exact values whenever suitable.*
☐	☒	For Bayesian analysis, information on the choice of priors and Markov chain Monte Carlo settings
☐	☒	For hierarchical and complex designs, identification of the appropriate level for tests and full reporting of outcomes
☐	☒	Estimates of effect sizes (e.g. Cohen's *d*, Pearson's *r*), indicating how they were calculated

Our web collection on statistics for biologists contains articles on many of the points above.

Software and code

Policy information about availability of computer code

Data collection	Publicly available datasets analyzed in this study were downloaded from the SRA and ENA databases using the SRAtoolkit (v2.10.5) fasterq-dump and wget commands respectively.
Data analysis	Integrated analysis of the novel and published datasets was performed using multiple publicly available tools in bash 4.2.46, Python 3.8.2 and R 4.0.3 environments. The following software tools were used, as illustrated in the methods: IsoSeq3(v3.4.0), CCS (v5.0.0), lima (v2.0.0), minimap2 (v2.17), sambamba (v0.5.6), Trim Galore (v0.6.6), STAR (v2.7.5b), TALON (v5.0), TranscriptClean (v2.0.2), SQANTI3 (v4.2), RSEM (v1.3), STARsolo (v2.7.9a), Seurat (v4.0.0), deepTools (v3.5.0), DESeq2 (v1.3.0), wiggletools (v1.2), CPAT (v3.0.2), Biostrings (v2.58), pfam_scan.pl (v1.6), HMMer (v3.3), RepeatMasker (v4.1.1), ChIPSeeker (v1.26.0), bedtools (v.2.29.2), bigWigAverageOverBed (v2), bowtie2 (v2.4.1), Genrich (v0.6), MACS2 (v2.1.0), DiffBind (v3.0.8), fgsea (v1.16.0), EdgeR (v3.32), Bismark (v0.22.3), methylKit (v1.16.0), liftOver (v9-Jul-2019), tximeta (v1.8.2), kallisto (v0.46.1), sleuth (v0.30.0), SUPPA2 (v2.3), gprofiler2 (v0.2.0), Mfuzz (v2.50.0), WGCNA (v1.69), miRanda (v3.3a), clusterProfiler (v3.18.0), ggnetwork (v0.5.10), HOMER (v4.10), TOBIAS (v0.11.1), VIPER (v1.24.0), DoRothEA (v1.2.2). All code has been deposited on GitHub at the following link: https://github.com/denis-torre/embryo-transcriptome

For manuscripts utilizing custom algorithms or software that are central to the research but not yet described in published literature, software must be made available to editors and reviewers. We strongly encourage code deposition in a community repository (e.g. GitHub). See the Nature Portfolio guidelines for submitting code & software for further information.

Data

Policy information about availability of data

All manuscripts must include a data availability statement. This statement should provide the following information, where applicable:

- Accession codes, unique identifiers, or web links for publicly available datasets
- A description of any restrictions on data availability
- For clinical datasets or third party data, please ensure that the statement adheres to our policy

The long- and short- RNA-Seq sequencing data have been deposited at GEO under the accession GSE190548. Uploaded data includes raw sequencing files for both data types, the novel human embryonic transcriptome in GTF format, as well as gene- and isoform-level counts across short-read RNA-Seq samples. Data can also be downloaded and interactively explored from the following resource website: https://denis-torre.github.io/embryo-transcriptome/. RNA-Seq reads were mapped to the GRCh38 reference genome, an the Ensembl v102 transcriptome assembly was used as a reference. The following publicly available datasets were further used for orthogonal validation of novel isoforms: Liu et al. (SRP163205), Yan et al. (SRP011546), Xue et al. (SRP018525), Petropoulos et al. (E-MTAB-3929), Mazid et al. (CNP0001454), Kagawa et al. (SRP323840), Mazin et al. (E-MTAB-6814), Xia et al. (PRJNA513257), Guo et al. (SRP028804), Wang et al. (SRP089891), Boroviak et al. (PRJEB29285).

Research involving human participants, their data, or biological material

Policy information about studies with human participants or human data. See also policy information about sex, gender (identity/presentation), and sexual orientation and race, ethnicity and racism.

Reporting on sex and gender	Embryos used in this study comprise both male and female sexes; gender is not proposed as a significant variable for the study. The results described herein apply to both sexes, and sex was not found to be a confounding variable in our results.
Reporting on race, ethnicity, or other socially relevant groupings	We did not include information on the race, ethnicity, or other socially relevant groupings related to the embryos used in this study, or to the patients who donated the embryos, as the embryos used were part of a retrospective biobank and we did not have the ability to control for those variables. We did not observe any confounding variables that could be related to such groupings in our results.
Population characteristics	Embryos were produced by patients undergoing IVF treatments in the IVF unit of Tel Aviv Sourasky Medical Center, Israel, between years 1997 and 2017. These patients reported to the unit with an indication of infertility. The samples used in this study are surplus preimplantation human IVF embryos at days 1-6 of development after fertilization. Spare embryos were donated by patients after completing their family fertility plan.
Recruitment	Patients reported to the IVF unit with an indication of infertility. Frozen embryos that were not needed for fertility use were donated for research with full informed consent and used in compliance with IRB following approval by the Israel National Ethics Committee (IRB 559/16). Mainly high/good quality embryos at the time of thawing were used for RNA preparation. Embryos were scored according to the routine IVF scoring system. Embryos were thawed according to their day of freezing (i.e. developmental day), and according to the study design aimed to extract RNA for an approximately equivalent number of embryos for each preimplantation developmental day.
Ethics oversight	Institutional Review Board of the Tel-Aviv Sourasky Medical Center, Israel, following approval by the Israeli National Ethics Committee.

Note that full information on the approval of the study protocol must also be provided in the manuscript.

Field-specific reporting

Please select the one below that is the best fit for your research. If you are not sure, read the appropriate sections before making your selection.

☒ Life sciences ☐ Behavioural & social sciences ☐ Ecological, evolutionary & environmental sciences

For a reference copy of the document with all sections, see nature.com/documents/nr-reporting-summary-flat.pdf

Life sciences study design

All studies must disclose on these points even when the disclosure is negative.

Sample size	Our aim was to map the isoform-annotated transcriptome of the human embryo through all preimplantation stages from the very early 1 cell stage until the blastocyst stage. For obvious reasons human embryos are difficult to obtain, and the very early developmental stages (1-4 cell stage) are even harder to achieve/extremely rare which inspired this collaboration. Specifically, spare IVF specimens in the last 2 decades are usually frozen at later stages (8 cell through blastocyst stage). That said, IVF embryos were donated for this research by patients only after completing their family fertility plan and following informed consent, which enabled us to get access to embryos that were frozen >20 years ago, at a time when spare IVF embryos were frozen at 1-4 cell stages. For that reason, we were able to conduct a robust study with significant sampling. The combined number of samples and total sequencing depth of our RNA-Seq data exceeds that of all comparable published studies combined (Yan et al., Xue et al., Liu et al.) for the earliest embryonic developmental stages (1C, 2C, 4C), and is comparable in size for the other profiled stages (8C, morula, blastocyst). We have also provided data on both short and long read sequencing platforms which enhances orthogonal analysis power. As such, the size our dataset is sufficient for publication.

Data exclusions	Donated embryos were thawed in the embryology lab, and those that did not survive thawing and failed quality control were excluded from further analyses. Four short-read RNA-Seq samples were excluded from the final study due to low read mapping rates. While a specific threshold for this value had not been previously set, these samples were also clear outliers using principal component analysis from gene expression data and were thus excluded.
Replication	Embryos were generated from 26 independent IVF cycles of 23 different patients. In order to ensure reproducibility of the experimental findings, our attempt was to extract RNA only from high quality embryos with the scoring system used during routine IVF clinical cycles. Nevertheless, it's important to note that human embryos are variable, thus although we aimed to select mainly high-quality embryos, in some stages (i.e. morula stage) we didn't have a large enough selection and used what we had access to as noted in our methods and analytics. Still, our collection is a very good presentation of IVF human preimplantation embryos, with its natural variability in the population.<br><br>Multiple published human embryo transcriptomic and epigenomic datasets were integrated to verify the validity of the findings in this study. 99% of the isoforms in our novel human embryonic transcriptome were indeed supported by short-read RNA-Seq from at least one of three integrated transcriptomic studies. In addition to this, the transcription start sites of the novel isoforms in the transcriptome were found to be significantly associated with euchromatin marks across multiple integrated epigenomic datasets, further supporting the validity of our findings.
Randomization	Samples were allocated into experimental groups based on their developmental stage at the time of RNA extraction. RNA Sequencing was performed in batches for different groups of samples over time; this was accounted for by providing batch information as a covariate to the statistical model performing the differential gene expression analysis.
Blinding	Blinding does not apply to this study. The developmental stage was determined based on embryo scoring prior to sequencing of each sample and was a key component of the analysis design.<br><br>With regards to collection, after signing an inform consent to donate the embryos for research, embryos were collected and thawed as a group according to their developmental stage and detached from any connection to identifying details. (i.e. the samples are anonymous/ blinded to the researches who extract RNA and prepare the samples for RNA-Seq and further analysis.)

Reporting for specific materials, systems and methods

We require information from authors about some types of materials, experimental systems and methods used in many studies. Here, indicate whether each material, system or method listed is relevant to your study. If you are not sure if a list item applies to your research, read the appropriate section before selecting a response.

Materials & experimental systems

n/a	Involved in the study
☒	☐ Antibodies
☒	☐ Eukaryotic cell lines
☒	☐ Palaeontology and archaeology
☒	☐ Animals and other organisms
☒	☐ Clinical data
☒	☐ Dual use research of concern
☒	☐ Plants

Methods

n/a	Involved in the study
☒	☐ ChIP-seq
☒	☐ Flow cytometry
☒	☐ MRI-based neuroimaging
